# The Irish Board of the College of Nursing

**Published:** 1917-10-27

**Authors:** Vera Matheson

**Affiliations:** Secretary. 23 Kildare Street, Dublin


*To the Editor of* The Hospital.

### The Irish Board of the College of Nursing.

*To the Editor of* The Hospital.

Sir,—There is at present so much uncertainty as to the scope and functions of two organisations which have recently arisen in Ireland to deal with questions connected with nursing, that we feel bound to draw public attention to the distinction between them.

The College of Nursing, Ltd., the membership of which already exceeds 7,000, was founded in 1916, and provides in its articles of association for a democratic control of the nursing profession by the members of that profession. Its objects are lofty and far-reaching, and at the same time thoroughly practical. It purposes to organise the nursing profession, to secure State Registration for the trained nurse, to raise and maintain the standard of training and establish a uniform curriculum, and to obtain professional advantages for the trained nurse.

Public-spirited Irish nurses and doctors saw at once that if Ireland were not included in the scope of the College, Irish nurses would be left in the lurch, and in the future would have to compete at a decided disadvantage with their British sisters, who would hold the certificate of the College. Upon their initiative, accordingly, an Irish Branch of the College was started in February of this year, which soon attracted in all parts of the country the support of those who were far-seeing enough to understand the best interests of the Irish nurse. It is admitted on every side that the training in Ireland is unequal, and that reform is needed. It is also agreed by all thinking people that by the very nature of her profession a nurse can never undertake to confine her life to one country. She never knows whither her work will lead her. Therefore an indispensable item of her equipment is a certificate which will carry weight all over the world. Such a certificate she will have if she joins the College of Nursing. The necessity of having an Irish Branch is therefore obvious.

This Irish Branch was fully formed and organised, and doing work, when another organisation appeared, with the title of "The Irish Nursing Board, approved by the Royal College of Surgeons in Ireland." In a circular-letter the Irish Nursing Board expresses the hope that it will "take the place in this country that the College of Nursing aims at taking in England." This it is quite impossible for it to do, because it is too narrow in conception. The College of Nursing is a world-wide movement, working for the benefit of English, Scotch, Welsh, Irish, and Colonial nurses, who, whatever their nationality or political opinions, are first and foremost nurses, with the needs and responsibilities of nurses. The Irish Nursing Board has started a Register, and proposes to grant a certificate; but this certificate can carry little weight outside Ireland, whilst, should State Registration be granted, the Irish Nursing Board can give no guarantee that its Register will be recognised by the State. With regard to raising the standard of training, an organisation which can promise no material benefit to the nurses cannot hope to effect abiding reform in the profession.

This being so, it is hoped that the public and the nurses will realise fully that in starting the Irish Nursing Board its promoters are working against the best interests of the Irish nurse, and retarding in no small degree the work of progression and reform.—I remain (on behalf of the Irish Board of the College of Nursing), yours faithfully,

Vera Matheson, Secretary.

23 Kildare Street, Dublin, October 19, 1917.

